# Occlusion of Internal Carotid Artery in Kimura's Disease

**DOI:** 10.1155/2010/407538

**Published:** 2010-03-04

**Authors:** Tomonori Tamaki, Node Yoji

**Affiliations:** Department of Neurosurgery, Tamanagayama Hospital, Nippon Medical School, Tokyo 206-8512, Japan

## Abstract

We describe a unique case of Kimura's disease in which cerebral infarction was caused by occlusion of the right internal carotid artery. A 25-year-old man with Kimura's disease was admitted to our hospital because of left hemiparesis. Computed tomography and magnetic resonance imaging of the head showed infarction in the right frontal and temporal lobes. Cerebral angiography demonstrated right internal carotid artery occlusion affecting the C1 segment, with moyamoya-like collateral vessels arising from the right opthalamic artery. Kimura's disease is a chronic disease characterized by the clinical triad of slowly enlarging subcutaneous masses with lymphoid hyperplasia in the head and neck. It often occurs in young Asian men. In our patient, the pathogenesis of internal carotid artery occlusion was unknown. There have only been a few case reports in which occlusion of the internal carotid artery was associated with autoimmune disease, and no previous cases of internal carotid occlusion associated with Kimura's disease have been reported. We suspected that occlusion of this patient's internal carotid artery may be caused by the autoimmune mechanism that underlies Kimura's disease.

## 1. Introduction

Kimura's disease is a chronic angiolymphoid proliferative disorder [1–3]. This disease shows male predominance and mainly occurs during the second and third decades of life. It is endemic to Asia, especially Japan and China. The chief clinical manifestation is asymptomatic unilateral soft tissue swellings, such as enlarged salivary glands and lymph nodes. Kimura's disease is associated with a variety of autoimmune diseases, including glomerulonephritis, Sjorgren's syndrome, and immunological disorders [1–3]. However, there have been no reports about Kimura's disease associated with cerebrovascular disease. We describe a patient with Kimura's disease who suffered from cerebral infarction caused by occlusion of the right internal carotid artery. We also discuss the mechanism of carotid occlusion in this patient and the relation between carotid artery occlusion and Kimura's disease.

## 2. Case Report

**Figure 1:**
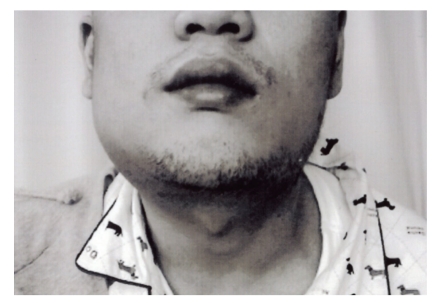
The photograph shows a mass protruding from the right neck.

**Figure 2:**
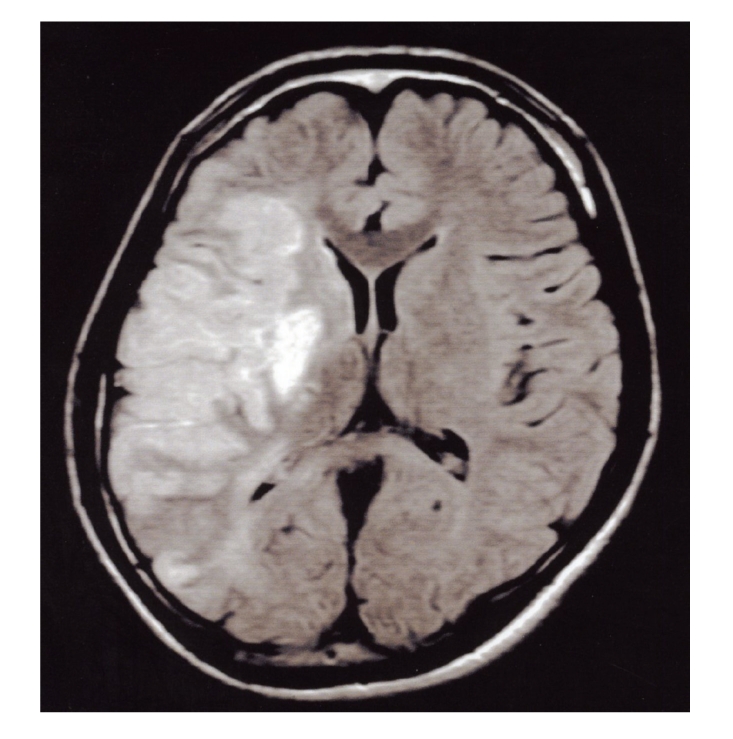
Axial fluid-attenuated inversion recovery magnetic resonance image showing cerebral infarction in the right cerebral hemisphere.

**Figure 3:**
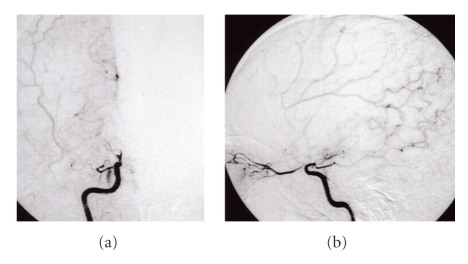
Right internal carotid artery angiogram. Angiogram reveals occlusion of the right internal carotid artery C1 segment. Note the collateral cerebral blood flow arising from posterior circulation.

A 25-year-old man was admitted to our hospital on May 5, 2006, after developing progressive left hemiparesis. Ten days before admission, he had noted an episode of numbness of the face and upper extremity on the left side. On examination, the patient was found drowsy and had dysarthria, right gaze, left-side hemianopia and left hemiparesis. Examination of cranial nerves showed mild left central-type facial weakness. The pupillary reflexes were normal to light. There were severe loss of pain, temperature sensation and proprioception of the patient's left-side body. There was a 4 × 4 cm soft subcutaneous mass behind the angle of the right mandibles. This mass was nontender and smooth, without any color change of the overlying skin (Figure 1). At admission, laboratory tests showed 25% eosinophils, and zinc sulfate turbidity was elevated to 13.7 U (normal range: 4.0–12.0 U). In addition, leukocyte alkaline phosphate was elevated to 432 IU/mL (normal range: 85–340 IU/mL). There was no elevation of tumor markers, such as carcinoembryonic antigen, carbohydrate antigen 19-9, or alpha-fetoprotein. The serum immunoglobulin E level was not measured. Serologic tests showed negative results for syphilis, antinuclear antibodies (ANA) and anti-DNA antibodies. Anticardiolipin antibodies (ACLs) and lupus anticoagulant were also negative. Coagulation studies disclosed normal values; prothrombin time was 13 seconds and activated partial thromboplastin time (APTT) was 35.3 seconds. Antithrombin III and protein C were within normal limits. Cerebrospinal fleuid (CSF) examination showed 1 cell/mm^3^, glucose 85 mg/dL, and protein level 55 mg/dL. Neither myelin basic protein nor oligoclonal band was present. In 1994, the patient had been treated for neck swelling at the Department of Otorhinolaryngology of Nippon Medical School Tamanagayama Hospital. Swellings were detected on both sides of the neck, and a diagnosis of Kimura's disease was established from histologic examination of the biopsy specimens. The patient was managed with conservative therapy. There was no family history of cerebrovascular or immunological disease. After admission, computed tomography and magnetic resonance imaging of the head showed infarction on the right frontal and temporal lobes (Figure 2). Single photon emission computed tomography of the brain showed a severe (about 70%) reduction of blood flow in the right cerebral hemisphere. Angiography disclosed occlusion of the right internal carotid artery at the C1 segment (Figure 3). Chest X-ray was normal. Transthoracic echocardiography and trans-esophagial echocardiography were normal, and the embolic source was not identified. Other causes of cranial vascular diseases were excluded by the clinical and laboratory workup. The patient was treated with conservative therapy and rehabilitation. Eight weeks after onset, he was transferred for rehabilitation. His last National Institute Health Stroke Scale (NIHSS) score was 18.

## 3. Discussion

Kimura's disease is a chronic angiolymphoid proliferative disorder. The cause of Kimura's disease remains unknown, but is thought to be related to allergy, because the patients often have eosinophilia and high serum immunoglobulin E levels [1, 3]. Complications such as atopic dermatitis, allergic rhinitis, asthma, and urticaria occur among patients with Kimura's disease [1, 3]. Kimura's disease is also associated with a variety of autoimmune diseases, including glomerulonephritis, arthritis, Sjögren's syndrome and immunological disorders [1, 3]. However, there have been no reports about cerebrovascular complications. In our patient, angiography revealed right internal carotid artery occlusion (C1 segment). Although the angiographic findings resembled those seen in moyamoya disease, there did differences from typical moyamoya disease. Our angiograms were not reveal bilateral C1 segment occlusion and typical moyamoya vessels. The pathogenesis of moyamoya disease is not understood, with genetic, autoimmune, and infectious causes all having been postulated [4–8]. Autopsy studies of patients with moyamoya disease classically show intimal thickening with crenulation of the elastic lamina, while inflammation is absent [4–8]. Hereditary factors and/or acquired disorders, for example, chronic autoimmune arteritis, may be involved in the occurrence of moyamoya disease, but the etiology and pathogenesis are unknown [4–8]. The mechanism leading to occlusion of the internal carotid artery in our patient is also unknown. However, there have been a few case reports in which occlusion of internal carotid artery was associated with autoimmune diseases, such as Takayasu's disease and systemic lupus erythematosus [4–8]. The inflammation and subsequent postinfectious autoimmune response associated with meningitis can lead to progressive vasculopathy that may represent a pathophysiologic mechanism for the arterial occlusions seen in moyamoya disease [5, 6]. Unfortunately, no cerebrovascular studies were done prior to the onset of Kimura's disease in our patient, so preexisting vascular disease cannot be excluded. In our patient, we suspected that occlusion of the internal carotid artery may be caused by the autoimmune mechanism that underlies Kimura's disease.
